# Predictors of COVID-19 Vaccination Among EMS Personnel

**DOI:** 10.5811/westjem.2022.4.54926

**Published:** 2022-07-11

**Authors:** Michael W. Hubble, Ginny K. Renkiewicz, Sandy Hunter, Randy D. Kearns

**Affiliations:** * Wake Technical Community College, Department of Emergency Medical Science, Raleigh, North Carolina; † Methodist University, Department of Health Care Administration, Fayetteville, North Carolina; ‡ The University of New Orleans, Department of Management & Marketing – College of Business Administration, New Orleans, Louisiana

## Abstract

**Introduction:**

Unvaccinated emergency medical services (EMS) personnel are at increased risk of contracting coronavirus disease 2019 (COVID-19) and potentially transmitting the virus to their families, coworkers, and patients. Effective vaccines for the severe acute respiratory syndrome coronavirus 2 virus exist; however, vaccination rates among EMS professionals remain largely unknown. Consequently, we sought to document vaccination rates of EMS professionals and identify predictors of vaccination uptake.

**Methods:**

We conducted a cross-sectional survey of North Carolina EMS professionals after the COVID-19 vaccines were widely available. The survey assessed vaccination status as well as beliefs regarding COVID-19 illness and vaccine effectiveness. Prediction of vaccine uptake was modeled using logistic regression.

**Results:**

A total of 860 EMS professionals completed the survey, of whom 74.7% reported receiving the COVID-19 vaccination. Most respondents believed that COVID-19 is a serious threat to the population, that they are personally at higher risk of infection, that vaccine side effects are outweighed by illness prevention, and the vaccine is safe and effective. Despite this, only 18.7% supported mandatory vaccination for EMS professionals. Statistically significant differences were observed between the vaccinated and unvaccinated groups regarding vaccine safety and effectiveness, recall of employer vaccine recommendation, perceived risk of infection, degree of threat to the population, and trust in government to take actions to limit the spread of disease. Unvaccinated respondents cited reasons such as belief in personal health and natural immunity as protectors against infection, concerns about vaccine safety and effectiveness, inadequate vaccine knowledge, and lack of an employer mandate for declining the vaccine. Predictors of vaccination included belief in vaccine safety (odds ratio [OR] 5.5, P=<0.001) and effectiveness (OR 4.6, P=<0.001); importance of vaccination to protect patients (OR 15.5, P=<0.001); perceived personal risk of infection (OR 1.8, P=0.04); previous receipt of influenza vaccine (OR 2.5, P=0.003); and sufficient knowledge to make an informed decision about vaccination (OR 2.4, P=0.024).

**Conclusion:**

In this survey of EMS professionals, over a quarter remained unvaccinated for COVID-19. Given the identified predictors of vaccine acceptance, EMS systems should focus on countering misinformation through employee educational campaigns as well as on developing policies regarding workforce immunization requirements.

## INTRODUCTION

As of August 2021, severe acute respiratory syndrome coronavirus 2 (SARS-CoV-2), the virus responsible for coronavirus disease 2019 (COVID-19), has infected more than 40 million Americans and is responsible for 649,299 deaths.^1^ The disease was classified by the World Health Organization (WHO) as a pandemic in March 2020, with more than 216 million cases and 4.5 million deaths reported globally as of August 2021.^2^ Among US healthcare workers (HCW) specifically, the Centers for Disease Control and Prevention (CDC) has reported 548,367 cases of COVID-19 and 1747 deaths.^3^

Due to frequent interactions with potentially infected patients, combined with the shortage of personal protective equipment at the time this study was conducted, emergency medical services (EMS) professionals are at particular risk of contracting as well as disseminating COVID-19. Besides their individual risk of acquiring COVID-19 in the workplace, EMS professionals may act as a vector and transmit the disease to susceptible patients, coworkers, friends, and family. For this reason, it is essential that all EMS professionals be vaccinated against COVID-19.

At the time this study was conducted, two mRNA vaccines received emergency use authorization (EUA) by the Food and Drug Administration (FDA) in December 2020, which demonstrated 94% and 95% efficacy against symptomatic COVID-19 in clinical trials among the general population.^4^,^5^ With respect to HCWs specifically, several studies demonstrated that vaccinating employees substantially reduced illness. Notably, Swift et al reported 78% and 96% vaccine effectiveness (VE) among 3210 partially and 44,011 fully vaccinated Mayo Clinic employees.^6^ Similarly, in a large-scale study of 23,324 HCWs in England, Hall et al realized a VE of 70% and 85% among partially and fully vaccinated employees, respectively.^7^

In addition to the mRNA vaccines, a viral vector vaccine was also granted EUA status February 27, 2021. The mRNA vaccines required a staggered two-injection process to achieve the most optimal results. While the single-dose viral vector vaccine did not offer the same protection against morbidity (66.3% in clinical trials),^8^ it did offer similar protection against mortality. Furthermore, the international Phase 3 data reported the vaccine was 85% effective in preventing severe cases of COVID-19. Of the 19,630 individuals who received the actual vaccine, there were three deaths reported, none related to either COVID-19 or the vaccine. Thus, the viral vector vaccine was deemed 100% effective in preventing COVID-19-related deaths in the study group.

Despite the protective benefits of vaccination, substantial vaccine hesitancy and resistance exists among the US general population, with 18% indicating that they are unlikely to accept the COVID-19 vaccine specifically.^9^ More importantly, overall vaccine hesitancy observed in the general population has been linked to the level of hesitancy among HCWs in general.^10^ To date, only two studies have explicitly addressed COVID-19 vaccine hesitancy and immunization rates of EMS personnel. In a cross-sectional survey of US firefighters and EMS personnel, Caban-Martinez et al reported that over half of their respondents were either uncertain or unlikely to receive the vaccine.^11^ However, this study was conducted prior to any issued EUA or formal vaccine approval by the FDA. A similar cross-sectional study conducted in Germany found a slightly higher willingness to receive the vaccine (57%), but this study was also conducted prior to widespread vaccine availability.^12^ Moreover, it is unclear whether these findings could be extrapolated to US EMS personnel.

*Population Health Research Capsule*

What do we already know about this issue?

*Unvaccinated EMS personnel are at increased risk of contracting and transmitting COVID-19.*

What was the research question?

*What is the vaccination rate of EMS professionals and what are the predictors of vaccination uptake?*

What was the major finding of the study?

*Nearly 25% of EMS personnel are unvaccinated against COVID-19, with contrasting opinions regarding vaccine safety and effectiveness.*

How does this improve population health?

*Our results suggest EMS systems should focus on countering misinformation through employee educational campaigns and developing policies regarding workforce immunization requirements.*

Given the lack of investigations of vaccine receptiveness of US EMS professionals in a post-vaccine era of COVID-19, we sought to document vaccination rates in a single state and identify predictors of vaccination uptake.

## METHODS

### Human Subject Review

Institutional review board approval for this study was obtained from Wake Technical Community College Department of Emergency Medical Science, and electronic informed consent was obtained from each respondent at the start of the survey.

### Instrument and Setting

We conducted a cross-sectional survey from April 27–May 18, 2021 to assess the attitudes, beliefs, and COVID-19 vaccination status of EMS personnel. Also included in the survey were illness profiles regarding COVID-19 illness and immunization for family, friends, coworkers, and the individual respondent. A unique, online survey was developed using constructs similar to the health belief model.^13^ Briefly, the health belief model posits that an individual’s assessment of their personal risk of illness, combined with their belief in the effectiveness of the recommended health behavior (eg, vaccination), predicts the likelihood of adopting the recommended behavior. Guided by these constructs, we designed the survey and then piloted it on a small group of EMS professionals. Based on the responses to the pilot, the survey was revised for clarity. The final survey consisted of 53 items and was designed to be completed within 10 minutes.

Links to the web-based survey (Qualtrics, Provo, UT) were emailed to EMS personnel listed as actively credentialed by the North Carolina Office of EMS via their data management vendor. Emergency medical technicians (EMT), advanced EMTs, and paramedic field professionals were invited to complete the survey. Due to variable and sometimes infrequent EMS responses and patient exposures, first responders certified at the emergency medical responder level were excluded from the survey. Participation was anonymous and voluntary, and no inducements to participate were provided.

### Statistical Methods

All data was exported from the Qualtrics web survey platform into a Microsoft Excel spreadsheet (Microsoft Corporation, Redmond, WA) and later imported into SPSS version 27 (IBM Corp., Armonk, NY) for analysis. All statistical analyses were two-tailed with statistical significance established at *P=*≤ 0.05.

Standard descriptive statistics were computed, and univariate comparisons for categorical variables were conducted using the chi square test, Fisher’s exact test, or Yate’s continuity correction as appropriate. We developed a multivariable binary logistic regression model to identify independent factors associated with uptake of the COVID-19 vaccine. All variables were entered into the model, and backward stepwise elimination was used to remove non-significant variables based on likelihood ratios. To evaluate model performance, we computed area under the curve of the receiver operating characteristic (AUC-ROC) for the final model.

## RESULTS

A total of 860 EMS professionals completed the survey in its entirety. Demographic and employment characteristics of respondents are shown in Table 1. The majority of respondents were male (66.5%), White (93.3%), paramedic credentialed (66.4%), employed full-time (78.3%), and held a college degree (64.1%). The average age of the respondents was 41.1 (± 12.4) years with a mean of 15.3 (±10.9) years of EMS experience. Of all respondents, only 582 (67.7%) had received the influenza vaccine during the 2020–2021 season, demonstrating some degree of underlying vaccine hesitancy in this sample.

Regarding the COVID-19 vaccine, 642 (74.7%) had already received or planned to soon receive the vaccine. The individual, familial, and coworker COVID-19 disease burden was extensive. A small yet significant portion of the sample (17.7%) had been previously infected, 23.4% lived in the same household as someone with a previous diagnosis, and over half (54.3%) had family members living outside the home with a prior occurrence of COVID-19 illness (Table 2). The vast majority (95.7%) knew at least one EMS coworker previously diagnosed with COVID-19.

In general, survey respondents reported that they believed the following: they are at higher risk for COVID-19 than the general population (67.1%); COVID-19 is a moderate to severe threat to the US population (68.7%); they had received enough information to make an informed decision about being immunized against COVID-19 (87.7%); the risk of side effects from the COVID-19 vaccines is outweighed by the prevention of the disease in the general public (71.7%); the vaccines are somewhat or very safe (77.1%) and effective (76.0%); it is important for HCWs to receive a COVID-19 vaccine to protect themselves (71.9%) and their patients (71.0%); and recalled their employer recommending a COVID-19 vaccine (76.6%). For each of these attitudes and beliefs, a univariate analysis observed significant differences between vaccinated and unvaccinated respondents (Table 2). In addition, respondents who received a seasonal influenza vaccination were also more receptive to vaccination for COVID-19 (80.2% vs 30.7%, *P*=<0.001).

Despite overall favorable opinions regarding vaccine safety and effectiveness, only 18.7% believed the COVID-19 vaccine should be mandatory for all EMS professionals, with most believing it should be optional (47.1%) or mandatory with the option to decline (34.2%), similar to the hepatitis B vaccination. Furthermore, respondents indicated a low level of trust in state government to take appropriate actions to reduce disease spread (41.9%) and opposition to any government actions that superseded individual objections to donning face masks while in public (45.3%).

Despite their belief that they were at greater risk of contracting COVID-19, few (43.7%) reported wearing a mask in the ambulance when not transporting a patient, or masking (29.4%) or practicing physical distancing (48.7%) while at the ambulance base. The lack of these risk-averting behaviors extended into public settings while off-duty, particularly among the unvaccinated (Table 2). The unvaccinated were also more comfortable with a member of their family being treated in a healthcare facility by unvaccinated HCWs (96.3%) or being treated and transported by unvaccinated EMS professionals (96.8%) compared to their vaccinated counterparts (66.7% and 67.8%, respectively).

The top reasons cited by respondents who did not receive one of the COVID-19 vaccines included concerns about safety and effectiveness, inadequate information to make an informed decision, concerns about vaccine side effects, reliance on the protective properties of personal health or natural immune response, and previous COVID-19 illness (Table 3). Of these, concern about vaccine safety was by far the most frequently cited reason for not accepting the vaccine (36.2%). For those respondents who did receive a COVID-19 vaccine, the most cited reasons for doing so included the desire to protect themselves, their families, and their patients; belief of increased work-related risk; seriousness of the disease; and the perception that benefits to vaccination outweighed the risks (Table 4).

Logistic regression odds ratios (OR), 95% confidence intervals (CI), and *P*-values for the prediction of vaccination uptake are shown in Table 5. The model adequately predicted COVID-19 vaccination uptake with an AUC-ROC of 0.96. Overall prediction accuracy of the model was 92.8% with a Hosmer and Lemeshow goodness of fit test (χ2 2.44, *P=*0.78), and Nagelkerke R^2^ 0.789. The factors retained in the final model included “previous receipt of influenza vaccine” (OR 2.57, *P*=0.003); “previously diagnosed with COVID” (OR 0.52, *P*=0.08); “perception of greater risk of COVID infection compared to general population” (OR 1.87, *P* 0.04); “positive belief in effectiveness of vaccine” (OR 4.63, *P*=<0.001); “positive belief in safety of vaccine” (OR 5.55, *P*=<0.001); “positive belief in importance of healthcare workers to receive the COVID-19 vaccine to protect their patients (OR 15.58, *P* =<0.001); and “received enough information to make an informed decision about being immunized against COVID-19” (OR 2.46, *P*=0.02).

## DISCUSSION

In 2019 the WHO listed 10 threats to global health; among these were vaccine hesitancy and a global pandemic.^14^ Alas, the world is now confronting both threats simultaneously. The rationale among the non-vaccinated is complicated, but misconceptions prevail regarding the safety and effectiveness of vaccines in general, and the COVID-19 vaccines specifically. The resulting suboptimal uptake of a safe and effective vaccine for an easily transmissible and potentially lethal infection has been christened the “pandemic public health paradox.”^15^ Unfortunately, HCWs, including EMS personnel, are not immune to the misinformation energizing vaccine hesitancy.

In our cross-sectional survey, we found a COVID-19 vaccination rate among EMS professionals in North Carolina of 74.7%, which is 55% higher than the national vaccination intention rate among US firefighters and EMS workers previously reported by Caban-Martinez et al.^11^ This proportion is also substantially greater than the previously reported influenza vaccination rates of North Carolina EMS professionals.^16^ Despite this, a substantial segment of the EMS workforce, their patients, families, and other contacts are still at considerable risk. The majority of survey respondents believed that COVID-19 posed a serious threat to public health, that they were at increased risk of work-related infection, and that the COVID-19 vaccines were safe and effective. However, these beliefs alone did not ensure a higher vaccination rate, and the contrasting opinions and beliefs between the vaccinated and unvaccinated were striking.

Among respondents, the reasons for receiving the COVID-19 vaccination were similar to results reported by Maltezou et al and included the motivation to protect themselves, their families, and their patients, as well as a desire to control the continued spread of a serious disease.^17^ Reasons for not receiving one of the vaccines included concerns about vaccine safety and effectiveness, insufficient knowledge of the vaccine, concerns with respect to side effects, prior COVID-19 infection, and reliance on personal health and natural immune response to combat any potential coronavirus disease. Similarly, Schrading et al also reported concerns about vaccine safety and effectiveness, side effects, and previous COVID-19 diagnosis as reasons for declining vaccination among a survey of US emergency department personnel.^18^ These concerns were echoed in a survey of HCWs at a large university healthcare system.^19^ Additional concerns cited by this healthcare system cohort included political involvement, vaccine research methodology, EUA (ie, a lack of full FDA approval), and the novelty of the vaccine.^19^

Our statewide survey was conducted during the period between the initial surge and the subsequent delta variant-fueled wave of the COVID-19 pandemic. During the data collection period, a statewide mandate for face coverings and social distancing in public settings was in place and daily infections were declining. There were 518–1988 daily cases reported in North Carolina during this time, and the cumulative COVID-19 cases ranged from 966,878 to 991,376.^20^ By the end of the survey, roughly 9.3% of the general population in the state had been diagnosed with COVID-19 compared to the 17.7% in our sample, highlighting the increased disease burden among EMS professionals. Whether this excess case rate was the result of true illness from work-related or off-duty exposures or a reflection of increased access to testing remains unknown. In addition to their own illness, most of the respondents reported either living in the same household as someone with a previous COVID-19 diagnosis (23.4%) or having family members living outside the home who had a similar diagnosis (54.3%).

In addition to some degree of vaccine hesitancy, our respondents also reported personal behaviors representing missed opportunities to reduce work-related disease transmission, such as wearing masks and physically distancing when possible while not actively engaged in patient care activities during their duty shift. These on-duty behaviors translated into off-duty behaviors, particularly among the unvaccinated, where most did not wear a mask or socially distance while in public settings despite an executive order issued by the governor of North Carolina mandating such preventive measures.

Because EMS professionals are crucial components of the healthcare system, maintaining wellness among this group is paramount, and it is incumbent upon EMS administrators to ensure a protected EMS workforce. Nonetheless, overcoming vaccine hesitancy is particularly problematic in the context of COVID-19 because of the unprecedented politicization of vaccine development and public health responses to the pandemic, as well as the unbridled spread of misinformation, especially via social media.

Several health beliefs expressed by our respondents are core constructs of various health behavior theories, which include the health belief model,^13^ the theory of reasoned action,^21^ and the multi-attribute utility model.^22^ Importantly, these beliefs represent targets for interventions for addressing vaccine hesitancy. Roughly half (50.5%) of respondents who listed a primary reason for remaining unvaccinated referred to vaccine misinformation including concerns about safety, effectiveness, side effects, acquiring COVID-19 illness from the vaccine itself, and general antivaccine sentiment (Table 3). These largely misinformation-based responses to vaccination may prove to be among the most difficult to overcome because broadly focused, information-based messaging alone is likely to be ineffective, particularly in light of the “backfire effect.” The backfire effect is the tendency of individuals to resist accepting evidence that conflicts with their beliefs and subsequently become even more entrenched in their acceptance of misinformation, which can exacerbate nescience in such situations.^23^ In addition, public health officials trying to educate the populace on mask wearing or other safety initiatives often issued confusing or contradictory information, leading to a lack of trust in the government to handle the pandemic properly.^24^ These ideas are supported in that only 6.4% of the unvaccinated attributed a lack of sufficient information as their primary reason for declining the vaccine.

Instead of broadly focused messaging, some observers recommend that the underlying emotions, beliefs, and attitudes be identified and that messaging strategies be tailored to these attitudes.^25^ Such strategies have included reporting the positive experience of vaccinated people to enhance overall trust in the vaccine^26, 27^; messaging that is people-centered and uses first-person accounts with emotional verbiage^28^; and the use of “trusted messengers” to disseminate information.^26^

Some have argued that the unvaccinated represent economic externalities and can therefore be addressed economically with both positive and negative financial incentives.^29^ Examples of positive incentives that have been used include gift cards, food, alcoholic beverages, lotteries, and scholarships, while negative incentives may include increased health insurance premiums for the unvaccinated and denied access to schools or retail spaces. However, these strategies have not been thoroughly evaluated and their effectiveness is unknown. It is likely that a subset of the unvaccinated will not be swayed by either incentives or messaging campaigns, a group that French et al dub the “active resistors,” who decline the vaccine based on strong personal, cultural, or religious beliefs.^30^ Unfortunately, few tools exist for increasing vaccine uptake in this group, although one possible strategy is a mandatory workplace vaccination policy.

Policies mandating influenza vaccination of HCWs have gained popularity in some settings due to low vaccine uptake. Such policies consistently yield influenza vaccine uptake rates above 90% while simultaneously providing for medical and religious exemptions.^31^ Similar policies for COVID-19 have been implemented for HCWs in some countries, including Greece and France.^32^ In the United States, compulsory COVID-19 vaccination of HCWs is supported by 68 professional organizations, including the American Medical Association, American Academy of Pediatrics, American College of Physicians, American College of Surgeons, American Public Health Association, and National League for Nursing,^33^ and has been implemented by many healthcare systems.^34^ Moreover, the National Association of EMS Physicians joined these organizations in calling for mandatory COVID-19 vaccination for EMS professionals.^35^

Resistance to mandatory vaccination was intense among our surveyed EMS professionals where only 18.7% of our total respondents supported a mandatory vaccination policy. Again, even within this overall low level of support for mandatory vaccination, the degree of divergence of opinions between vaccinated and unvaccinated was stark. Mandatory vaccination was supported by 25.1% of the vaccinated respondents compared to 0.0% of the unvaccinated group. A total of 294 (34.2%) respondents overall supported an alternative policy to make COVID-19 vaccination mandatory for EMS professionals, with a declination option similar to most policies addressing the hepatitis B vaccine. Overall, nearly half (47.1%) believed that COVID-19 vaccination should be entirely optional. Comparatively, in a similar survey of North Carolina paramedics regarding compulsory influenza vaccination, 52.3% believed vaccination should be entirely optional, 38.7% supported mandatory vaccination with the option to decline, and 9.1% agreed with compulsory vaccination.^16^ Thus, it appears that opposition to mandatory COVID-19 vaccination is similar to that of influenza vaccination and has remained consistent over time among North Carolina EMS professionals. Consequently, although the feasibility and true impact of implementing such a strategy in EMS systems is unknown, resistance to a mandatory COVID-19 immunization policy in any form should be anticipated.

## LIMITATIONS

This study has several notable limitations, and our results should be interpreted accordingly. First, our survey was web-based, voluntary, and subject to the usual response and recall biases, and the cross-sectional nature of the data prevented us from drawing any causal inference between attitude and belief variables and COVID-19 vaccine acceptance. Additionally, the survey invitation was emailed by the North Carolina Office of EMS via their data management vendor to ensure the provision of anonymity. The exact number of personnel who received the link is unknown. Thus, it isn’t possible to calculate a survey response rate.

Our sample was comprised entirely of North Carolina EMS personnel and the generalizability of our findings to EMS professionals outside of North Carolina is unknown. Furthermore, the data was collected prior to the delta or omicron variants becoming the predominant circulating strain. The EMS vaccination rates may have since been influenced by the extensive attention given by public health officials to this strain of COVID-19 and its accompanying surge in cases, hospitalizations, and deaths.

Our survey did not specifically question respondents regarding understanding of or acceptance of one vaccine type vs another (mRNA vs viral vector). Nor did our survey specifically look at acceptance as it related to convenience, one dose vs two, or storage and distribution factors for the mRNA vaccines. Any targeted messaging campaign created to increase vaccine uptake should consider these variables and provide additional information as appropriate. Lastly, this survey was sent to EMS professionals who were active on an EMS agency roster. We did not survey those who were in other medical fields, educators, or those who may have been between jobs.

## CONCLUSION

In this cross-sectional survey of North Carolina EMS professionals, COVID-19 vaccination rates were higher than have been previously reported, but a substantial subset remain at risk. Previous influenza vaccination, a perception of an increased risk for contracting the illness, sense of duty to protect patients, adequate information for decision-making, prior COVID-19 diagnosis, and favorable beliefs about vaccine safety and effectiveness were all predictive of vaccination acceptance. Nonetheless, erroneous beliefs and vaccine safety and effectiveness concerns were extensive, and resistance to mandatory vaccination was fervent. Notably, concern about safety was the most frequently cited reason for not accepting the COVID-19 vaccine. The EMS systems should focus their efforts on combating misinformation through strategically targeted employee educational campaigns as well as developing policies regarding immunization requirements and comprehensive workplace safety practices.

## Figures and Tables

**Table 1:** Sociodemographic characteristics of respondents.

Parameter	N = 860 n (%)
COVID-19 Vaccination Status	
Have received or plan to receive	642 (74.7%)
Do not plan to receive	218 (25.3%)
Age (mean [SD])	41.1 (12.4)
Male Gender	572 (66.5%)
Race	
White	802 (93.3%)
Black	16 (1.9%)
Multi-racial	26 (3.0%)
Native American	10 (1.2%)
Asian American or Pacific Islander	6 (0.7%)
Hispanic Origin	28 (3.3%)
Employed fulltime	673 (78.3%)
Level of EMS certification	
EMT	224 (26.0%)
Advanced EMT	65 (7.6%)
Paramedic	571 (66.4%)
Years of EMS experience (mean [SD])	15.3 (10.9)
Highest level of education in any field	
High school	45 (5.2%)
Some college	264 (30.7%)
AAS degree	243 (28.3%)
Bachelor’s degree	222 (25.8%)
Master’s degree	77 (9.0%)
Doctoral degree	9 (1.0%)

*COVID-19*, coronavirus disease of 2019; *SD*, standard deviation; *EMS*, emergency medical service; *EMT*, emergency medical technician; *AAS*, associate of applied science.

**Table 2:** Differences between vaccinated and unvaccinated respondents.

Respondent Characteristic	All respondents N (%)	Unvaccinated N (%)	Vaccinated N (%)	P-value
Received influenza vaccine for 2020–2021 season	582 (67.7%)	67 (30.7%)	515 (80.2%)	<0.001
Previously diagnosed with COVID-19	152 (17.7%)	52 (23.9%)	100 (15.6%)	<0.008
Someone in same household previously diagnosed with COVID-19	201 (23.4%)	67 (30.7%)	134 (20.9%)	<0.004
Family member(s) living outside respondent’s household previously diagnosed with COVID-19	467 (54.3%)	124 (56.9%)	343 (53.4%)	0.420
Friend(s) previously diagnosed with COVID-19	726 (84.4%)	179 (82.1%)	547 (85.2%)	0.327
Coworker(s) previously diagnosed with COVID-19	823 (95.7%)	207 (95.0%)	616 (96.0%)	0.665
Has cared for anyone ill with COVID-19 while performing duties as an EMS professional	782 (90.9%)	206 (94.5%)	576 (89.7%)	0.047
Feel my level of risk is higher than the general population for getting COVID-19	577 (67.1%)	85 (39.0%)	492 (76.6%)	<0.001
Agree the COVID-19 vaccine is somewhat or very effective	654 (76.0%)	43 (19.7%)	611 (95.2%)	<0.001
Agree the COVID-19 vaccine is somewhat or very safe	663 (77.1%)	48 (22.0%)	615 (95.8%)	<0.001
Agree or strongly agree it is important for healthcare workers to receive the COVID-19 vaccine to protect themselves	618 (71.9%)	23 (10.6%)	595 (92.7%)	<0.001
Agree or strongly agree it is important for healthcare workers to receive the COVID-19 vaccine to protect their patients	611 (71.0%)	20 (9.2%)	591 (92.1%)	<0.001
Received training or education material from employer on the COVID-19 vaccine or COVID-19 illness	726 (84.4%)	184 (84.4%)	542 (84.4%)	0.285
Recall of employer recommending COVID-19 vaccine	659 (76.6%)	129 (59.2%)	530 (82.6%)	<0.001
Wears a mask in the ambulance when not transporting a patient	376 (43.7%)	84 (38.5%)	292 (45.5%)	0.125
Wears a mask at the ambulance base between calls	253 (29.4%)	38 (17.4 %)	215 (33.5%)	<0.001
Socially distances at the ambulance base between calls	419 (48.7%)	66 (30.3%)	353 (55.0%)	<0.001
Wears a mask in public while off-duty	632 (73.5%)	89 (40.8%)	543 (84.6%)	<0.001
Socially distances in public while off-duty	644 (74.9%)	100 (45.9%)	544 (84.7%)	<0.001
Received enough information to make an informed decision about being immunized against COVID-19	754 (87.7%)	171 (78.4%)	583 (90.8%)	<0.001
Would be comfortable if a member of my family were being treated in a healthcare facility by healthcare workers unvaccinated against COVID-19	574 (66.7%)	210 (96.3%)	364 (56.7%)	<0.001
Would be comfortable if a member of my family were being transported by ambulance and cared for by EMS professionals who have not been vaccinated against COVID-19	583 (67.8%)	211 (96.8%)	372 (57.9%)	<0.001
Has previously reported to work despite experiencing cold or flu-like symptoms or those symptoms that could be precursors to COVID-19	224 (26.0%)	59 (27.1%)	165 (25.7%)	0.759
Agree or strongly agree the risk of side effects from the COVID-19 vaccine is outweighed by the prevention of the disease in the general public	617 (71.7%)	74 (33.9%)	543 (84.6%)	<0.001
Believes that COVID-19 is a moderate to severe threat to the US population as a whole	591 (68.7%)	53 (24.3%)	538 (83.8%)	<0.001
Trusts state government to take the appropriate actions to reduce the spread of COVID-19	360 (41.9%)	18 (8.3%)	342 (53.3%)	<0.001
Believe my state government should prioritize reducing the spread of COVID-19 over individual objections to mask mandates	470 (54.7%)	33 (15.1%)	437 (68.1%)	<0.001
Believes the COVID-19 vaccine				
Should not be mandatory for all EMS workers	405 (47.1%)	206 (94.5%)	199 (31.0%)	<0.001
Should be mandatory for all EMS workers, but with option to decline	294 (34.2%)	12 (5.5%)	282 (43.9%)	
Should be mandatory for all EMS workers	161 (18.7%)	0 (0.0%)	161 (25.1%)	

*COVID-19*, coronavirus disease of 2019; *EMS*, emergency medical services;

**Table 3:** Primary reason why respondents did not receive COVID-19 vaccination

Reason	N (%)
I am concerned about the safety of the vaccine.	79 (36.2%)
I don’t think the COVID-19 vaccine is effective.	16 (7.3%)
I have not received enough information about the COVID-19 vaccine to make a decision.	14 (6.4%)
I am worried about the side effects of the COVID-19 vaccine.	12 (5.5%)
I’m healthy and don’t worry about getting COVID-19.	11 (5.0%)
I have had COVID-19 and don’t think I will get COVID-19 again.	11 (5.0%)
I don’t consider COVID-19 to be a serious illness.	9 (4.1%)
My natural immune system will protect me.	7 (3.2%)
It is not required by my employer.	7 (3.2%)
I don’t consider myself to be in a targeted group for which immunization is recommended.	4 (1.8%)
Religious reasons	4 (1.8%)
I am generally against vaccines.	2 (0.9%)
I believe the flu vaccine gave me the flu and I fear the COVID-19 vaccine may give me COVID-19.	1 (0.5%)
I have had a flu vaccine before and got sick anyway and would expect the same from the COVID-19 vaccine.	1 (0.5%)
I am allergic to the vaccine.	1 (0.5%)
Other	39 (17.9%)

**Table 4:** Reasons why respondents accepted COVID-19 vaccine COVID-19, coronavirus disease of 2019.

Reason	N (%)
Being vaccinated protects my family.	79 (36.2%)
I feel I am at risk for COVID-19 because of my work.	16 (7.3%)
I think it protects me from getting COVID-19.	14 (6.4%)
COVID-19 is a serious disease.	12 (5.5%)
I don’t want to expose my family to COVID-19 should I become infected at work.	11 (5.0%)
The benefits of the COVID-19 vaccine outweigh the risk of any side effects.	11 (5.0%)
Being vaccinated protects my patients.	9 (4.1%)
I work with patients at risk of complications from COVID-19, and I don’t want to expose them to COVID-19.	7 (3.2%)
My employer provides free COVID-19 vaccination.	7 (3.2%)
I will miss fewer days of work due to illness.	4 (1.8%)
I’ve had the flu in the past and don’t want to experience COVID-19.	4 (1.8%)
I was encouraged by my personal physician.	2 (0.9%)
I have a health condition (eg, heart disease, pulmonary disease) that might be exacerbated if I got COVID-19.	1 (0.5%)
I was encouraged by my coworkers.	1 (0.5%)
Other	1 (0.5%)

*COVID-19*, coronavirus disease of 2019

**Table 5:** Logistic regression model results for prediction of COVID-19 vaccination.

Parameter	Estimate (B)	Odds ratio (95% CI)	P value
Did you receive the influenza vaccine during last year’s influenza season? (reference category = “no”)	0.946	2.57 (1.37–4.81)	0.003
Previously diagnosed with COVID (reference category = “no”)	−0.648	0.52 (0.25–1.08)	0.081
Perception of greater risk of COVID infection compared to general population (reference category = “perceived risk less than or equal to general population”)	0.626	1.87 (1.01–3.46)	0.047
Positive belief in effectiveness of vaccine (reference category = “not at all effective or not very effective”)	1.534	4.63 (2.20–9.76)	< 0.001
Positive belief in safety of vaccine (reference category = “not at all safe” or “not very safe”)	1.715	5.55 (2.61–11.79)	< 0.001
Positive belief in importance of healthcare workers to receive the COVID-19 vaccine to protect their patients. (reference category = “strongly disagree” or “disagree”)	2.746	15.58 (7.74–31.33)	< 0.001
Have you received enough information to make an informed decision about being immunized against COVID-19? (reference category = “no”)	0.903	2.46 (1.12–5.39)	0.024

*COVID-19*, coronavirus disease of 2019.

## References

[1] Centers for Disease Control and Prevention (2012). Updated Recommendations for Use of Tetanus Toxoid, Reduced Diphtheria Toxoid, and Acellular Pertussis (Tdap) Vaccine in Adults Aged 65 Years and Older — Advisory Committee on Immunization Practices (ACIP), 2012. MMWR Morb Mortal Wkly Rep.

[2] Miller T, Olivieri P, Singer E (2017). The utility of Tdap in the emergency department. Am J Emerg Med.

[3] Pacella C (2021). Occupational exposures, infection control, and standard precautions.

[4] Buenger LE, Webber EC (2020). Clinical decision support in the electronic medical record to increase rates of influenza vaccination in a pediatric emergency department. Pediatr Emerg Care.

[5] Casalino E, Ghazali A, Bouzid D (2018). Emergency department influenza vaccination campaign allows increasing influenza vaccination coverage without disrupting time interval quality indicators. Intern Emerg Med.

[6] Ozog N, Steenbeek A, Curran J (2020). Attitudes toward influenza vaccination administration in the emergency department among patients: a cross-sectional survey. J Emerg Nurs.

[7] Harris Health System (2021). Harris Health Facts and Figures.

[8] Mackey K, Ayers CK, Kondo KK (2021). Racial and ethnic disparities in COVID-19-related infections, hospitalizations, and deaths: a systematic review. Ann Intern Med.

[9] Houston Health Department (2019). Health Disparity and Health Inequity: 2019 Trends and Data Report Houston/Harris County Summary Report.

[10] Ordonez E, Dowdell K, Navejar NM (2021). An assessment of the social determinants of health in an urban emergency department. West J Emerg Med.

[11] Harris County Public Health (2021). COVID-19 Vaccine Distribution.

[12] Kwok KO, Lai F, Wei WI (2020). Herd immunity – estimating the level required to halt the COVID-19 epidemics in affected countries. J Infect.

[13] Hotez PJ (2020). COVID-19 meets the antivaccine movement. Microbes Infect.

[14] Kaiser Family Foundation (2021). Early state vaccination data raise warning flags for racial equity.

[15] Kinder Institute for Urban Research (2021). Mapping inequity in Houston’s COVID-19 vaccination rollout.

[16] Romer D, Jamieson KH (2020). Conspiracy theories as barriers to controlling the spread of COVID-19 in the U.S. Soc Sci Med.

[17] Morley C, Unwin M, Peterson GM (2018). Emergency department crowding: a systematic review of causes, consequences and solutions. PLoS One.

[18] Lin C, Tu P, Beitsch LM (2020). Confidence and receptivity for COVID-19 vaccines: a rapid systematic review. Vaccines (Basel).

[19] Gamble VN (1997). Under the shadow of Tuskegee: African Americans and health care. Am J Public Health.

[20] Skloot R (2011). The Immortal Life of Henrietta Lacks.

[21] Khubchandani J, Macias Y (2021). COVID-19 vaccination hesitancy in Hispanics and African-Americans: a review and recommendations for practice. Brain Behav Immun Health.

[22] Shen MJ, Peterson EB, Costas-Muñiz R (2018). The effects of race and racial concordance on patient-physician communication: a systematic review of the literature. J Racial Ethn Health Disparities.

